# New York Letter

**Published:** 1882-12

**Authors:** 


### Domestic Correspondence.

---

### ARTICLE III.

### NEW YORK LETTER.

[The following was accidentally omitted from the letter of our New York correspondent published in our last issue.—ED.]

You have all heard of the terrible Seguin tragedy. It has at least one more cheerful side. It has brought out the most heartfelt expressions of sympathy from all the members of the profession here. Every society has passed resolutions of condolence, and expressed the hope that Dr. Seguin will not withdraw from his work here, where he has taken so high a position. He will go abroad for the present.

The post graduate schools have opened, and are all doing well. The number of pupils is not very large—twenty to thirty—but it will doubtless increase.

We are to have a new weekly medical journal. The New York *Medical Journal and Obstetrical Review* is to drop its obstetrical appendix and come forth as a medical weekly. Dr. Foster, who will continue the editorship, is an excellent editor, though not a very strong writer.

Dr. Marion Sims gave a grand reception to Dr. Gross, of Philadelphia, recently. It was held at the Brunswick Hotel, and was a sumptuous affair.

---

A SEWER is being constructed through an old cemetery in this city, bringing human remains to the surface, with the possibilities [illegible] all sorts of infectious diseases.

